# Trends in where people buy their vaping products and differences by user and device characteristics: A population study in England, 2016–23

**DOI:** 10.1111/add.16387

**Published:** 2023-11-14

**Authors:** Sarah E. Jackson, Harry Tattan‐Birch, Jamie Brown

**Affiliations:** ^1^ Department of Behavioural Science and Health University College London London UK; ^2^ SPECTRUM Consortium London UK

**Keywords:** Disposable vapes, e‐cigarettes, on‐line, population survey, supermarkets, vape shops

## Abstract

**Aims:**

To explore where people in England typically buy e‐cigarettes/vaping products, how their characteristics and the types of products purchased differ according to the source of purchase and whether this is changing over time.

**Design:**

This was a nationally representative monthly cross‐sectional survey.

**Setting:**

The study was conducted in England, 2016–23.

**Participants:**

A total of 6507 adults (≥ 18 years) who reported current vaping.

**Measurements:**

Participants were asked where they usually buy their vaping products: vape shops, supermarkets/convenience stores, on‐line or other sources (‘other’ or cheap from friends).

**Findings:**

Up to 2020, vape shops were the most common source of purchase (mean monthly proportion = 43.3%), followed by supermarkets/convenience stores (29.7%) and on‐line retailers (20.5%). In 2020, these purchasing patterns shifted: on‐line purchasing increased and vape‐shop purchasing fell. The rise in on‐line purchasing was short‐lived, peaking at 33.6% [95% confidence interval (CI) = 31.0–36.5%] in July 2021, and soon returned to baseline levels. However, the fall in vape‐shop purchasing persisted, remaining below 31% from July 2021 onwards, displaced by a rapid rise in purchasing from supermarkets and convenience stores from 31.6% (95% CI = 29.6–33.7%) in January 2021 to 48.5% (95% CI = 42.9–54.8%) by April 2023. This rise was most pronounced among younger adults (aged 18–35 years; *P*_interaction_ < 0.001) and those mainly using disposable devices (*P*_interaction_ = 0.013). Purchasing from other sources was relatively rare, and declined from 9.1% (95% CI = 6.6–12.7%) in July 2016 to 4.3% (95% CI = 2.6–7.1%) in April 2023.

**Conclusions:**

Supermarkets and convenience stores have recently overtaken vape shops to become the most popular places to buy vaping products in England. This change appears to have been driven by the rising popularity of new disposable e‐cigarettes among younger adults, who tend to buy these products from supermarkets/convenience stores.

## INTRODUCTION

E‐cigarettes have rapidly gained popularity in the past decade, particularly among people who are trying to quit or reduce their smoking [1]. In England, e‐cigarettes are widely available from specialist ‘vape shops’, supermarkets, convenience stores and on‐line, with a variety of different devices and nicotine strengths available. It is important to know where people usually buy their e‐cigarettes/vaping products, how the products and users’ characteristics vary by the source of purchase and how this is changing over time. This can inform policies that encourage the use of e‐cigarettes as a harm reduction tool while discouraging uptake among young people who have never smoked.

Representative surveys have provided some data on source of purchase of e‐cigarettes among adults in England. In the 2016 International Tobacco Control Four Country Smoking and Vaping Survey (*n* = 667), the preferred location for purchasing vaping products and supplies was fairly evenly distributed among vape shops (30.2%), on‐line (32.1%) and other retailers (37.7%; including supermarkets, convenience stores, cross‐border purchases and other sources) [2]. In the Smoking Toolkit Study between 2016 and 2020 (*n* = 3786), vape shops were the most common source of purchase (42.1%), followed by supermarkets (16.8%), on‐line vaping retailers (13.0%), newsagents (11.8%), other on‐line retailers (6.8%), petrol stations (2.0%) and friends (1.8%) [3]. In the latter study, there was some indication that this pattern was changing, with supermarkets cutting into vape shops’ share of the market [3]. Beyond these headline figures, relatively little is known about who is buying their products from where, what types of products they are buying and how this has changed over recent years in the context of the COVID‐19 pandemic and an evolving e‐cigarette market.

This study therefore aimed to provide a comprehensive update on where people in England buy their vaping products, analyse associations with user and product characteristics and explore changes over time. Specific research questions were:

1. What proportion of adults in England who vape report mainly buying their vaping products from:
  - vape shops;
  - on‐line vape retailers;
  - supermarkets/convenience stores;
  - other retailers?
2. How does source of purchase differ according to socio‐demographic characteristics and smoking status?
3. How does source of purchase differ according to main type of e‐cigarette used, usual nicotine concentration and vaping frequency?
4. To what extent have adults’ main source of vaping products, and differences between subgroups of vapers, changed between 2016 and 2023?

## METHOD

### Pre‐registration

The study protocol and analysis plan were pre‐registered on Open Science Framework (osf.io/85tk3/). We made two amendments. The first was to include ‘do not know’ as an additional category for nicotine concentration in the regression analyses. We had originally planned to exclude these responses, but we observed considerable differences by source of purchase (see Table 1), which seemed important to explore further. The second was to test interactions between each user and device characteristic and survey month without adjustment for other characteristics, as we encountered issues with model convergence.

**TABLE 1:** Descriptive data on source of purchase, overall and by user and device characteristics (across all survey waves; *n* = 6507).

		Usual source of purchase of vaping products			
	Unweighted *n*	Vape shop	Supermarket/convenience store	On‐line	Other
Overall	6507	37.3%	32.3%	24.4%	5.9%^a^
Age (years)					
18–24	1020	41.0%	31.9%	22.6%	4.6%
25–34	1453	40.1%	30.3%	24.3%	5.4%
35–44	1160	36.3%	30.9%	27.5%	5.2%
45–54	1219	36.2%	33.2%	24.1%	6.5%
55–64	980	34.6%	34.3%	24.5%	6.7%
65+	675	30.6%	38.2%	21.7%	9.4%
Gender					
Men	3514	36.6%	30.2%	27.0%	6.3%
Women	2962	38.3%	34.8%	21.4%	5.5%
In another way	30	27.1%	52.8%	16.7%	3.3%
Social grade C2DE					
ABC1	3500	35.0%	31.2%	28.8%	4.9%
C2DE	3007	39.2%	33.3%	20.7%	6.8%
Smoking status					
Never smoking	408	29.3%	34.2%	30.3%	6.2%
Former smoking	2577	35.5%	28.7%	30.0%	5.8%
Current smoking	3522	39.6%	34.8%	19.6%	6.0%
Device type					
Disposable	591	16.7%	64.4%	11.5%	7.4%
Refillable	4827	42.2%	25.4%	26.5%	5.8%
Pod	1025	26.1%	46.5%	22.4%	5.1%
Nicotine concentration					
No nicotine	815	35.5%	30.3%	26.3%	7.9%
6 mg/ml or less	2617	43.1%	24.7%	27.3%	4.9%
7–11 mg/ml	746	31.8%	41.3%	21.0%	5.9%
12–19 mg/ml	1458	36.6%	35.7%	21.9%	5.8%
20 mg/ml or more	455	29.2%	42.1%	22.0%	6.6%
Do not know	357	22.4%	48.6%	20.5%	8.5%
Frequency of vaping					
Non‐daily	1362	35.2%	36.9%	21.1%	6.9%
Daily	4453	38.7%	31.0%	24.9%	5.4%

*Notes*: Data shown are weighted row percentages. Note that there were missing data for some variables (gender *n* = 1; device type *n* = 64; nicotine concentration *n* = 59; frequency of vaping *n* = 692), so numbers do not sum to the total sample size for these variables. Valid percentages are shown for ease of interpretation. See Supporting information, Tables S2–S5 for data stratified by survey year (2016/2017, 2018/2019, 2020/2021, 2022/2023).

^a^ 1.2% ‘cheap from friends’, 4.7% ‘other’.

### Design

Data were drawn from the ongoing Smoking Toolkit Study, a monthly cross‐sectional survey of a representative sample of adults in England [4]. The study uses a hybrid of random probability and simple quota sampling to select a new sample of approximately 1700 adults each month. Comparisons with other national surveys and sales data indicate that key variables such as socio‐demographic characteristics, smoking prevalence and cigarette consumption are nationally representative [4, 5].

Data were initially collected through face‐to‐face computer‐assisted interviews. However, social distancing restrictions under the COVID‐19 pandemic meant that no data were collected in March 2020, and data from April 2020 onwards have been collected via telephone. The telephone‐based data collection uses broadly the same combination of random location and quota sampling and weighting approach as the face‐to‐face interviews, and comparisons of the two data collection modalities indicate good comparability [6, 7, 8].

Since April 2022, questions regarding source of purchase and device characteristics have only been assessed quarterly (in April, July, October and January) due to availability of competitive research funding.

For the present study, we used aggregated data from respondents to the survey during the period from July 2016 (the first wave in which detailed e‐cigarette usage characteristics were first recorded) to April 2023 (the most recent wave to assess source of purchase available at the time of analysis). Waves in which source of purchase was not assessed (May/June/August/September/November/December 2022, February/March 2023), since the switch from monthly to quarterly assessment of these variables, were excluded. We restricted our sample to those aged ≥ 18 years who reported current vaping (i.e. reported currently using e‐cigarettes to cut down on the amount they smoke, in situations when they are not allowed to smoke, to help them stop smoking or for any other reason).

### Measures

#### Source of purchase

Participants who vape were asked: ‘From where do you usually buy your disposable e‐cigarette or vaping device, pre‐filled cartridges, e‐liquids or electronic cigarette?’. Response options included the following types of retailer:

- Vape shop—‘specialist vape/electronic cigarette retailer—not on‐line’
- On‐line vape retailer—‘specialist vape/electronic cigarette retailer—on‐line’
- Other on‐line retailer—‘other on‐line retailer’
- Newsagent—‘newsagent/off licence/corner shop’
- Petrol station—‘petrol garage shop’
- Supermarket—‘supermarket’
- Friend—‘buy them cheap from friends’
- Other—‘other’

Participants could select one option, reflecting where they usually bought their vaping products. The question did not specify a time‐frame for usual source of purchase. For our analyses, we classified the responses into four categories:

- Vape shop (response a)
- Supermarket/convenience store (d–f)
- On‐line (b and c)
- Other (g and h)

#### Socio‐demographic characteristics

Age, gender and occupational social grade were recorded. Age was analysed as a categorical variable (18–24, 25–34, 35–44, 45–54, 55–64 and ≥ 65 years), with groups collapsed to 18–34 (younger), 35–64 (middle‐aged) and ≥ 65 (older) for tests of interactions. In each case, the youngest age group was used as the reference category. Gender was self‐reported as man, woman or in another way. The latter category was excluded from regression analyses due to low numbers. Occupational social grade was categorized as ABC1 (managerial, professional and upper supervisory occupations) and C2DE (manual routine, semi‐routine, lower supervisory and long‐term unemployed).

#### Smoking status

Participants were asked which of the following best applies to them:

- ‘I smoke cigarettes (including hand‐rolled) every day’
- ‘I smoke cigarettes (including hand‐rolled), but not every day’
- ‘I do not smoke cigarettes at all, but I do smoke tobacco of some kind (e.g. pipe, cigar or shisha)’
- ‘I have stopped smoking completely in the last year’
- ‘I stopped smoking completely more than a year ago’
- ‘I have never been a smoker [i.e. (have never) smoked for a year or more]’

Responses (a–c) were considered current smoking, (d) or (e) former smoking and (f) never (regular) smoking.

#### Main type of e‐cigarette used

Device type was assessed with the question: ‘Which of the following do you mainly use …?’ Response options were:

- Disposable—‘a disposable e‐cigarette or vaping device (non‐rechargeable)’
- Refillable—‘an e‐cigarette or vaping device with a tank that you refill with liquids (rechargeable)’ or ‘a modular system that you refill with liquids (you use your own combination of separate devices: batteries, atomizers, etc.)’
- Pod—‘an e‐cigarette or vaping device that uses replaceable pre‐filled cartridges (rechargeable)’

#### Usual nicotine concentration

Nicotine concentration was assessed with the question: ‘Does the electronic cigarette or vaping device you mainly use contain nicotine?’, with response options ‘yes’, ‘no’ and ‘do not know’. Those who responded ‘yes’ to this question were asked: ‘What strength is the e‐liquid that you mainly use in your electronic cigarette or vaping device?’. We reported descriptive data on the following response categories:

- No nicotine
- 6 mg/ml (~ 0.6%) or less
- 7 mg/ml (~ 0.7%) to 11 mg/ml (~ 1.1%)
- 12 mg/ml (~ 1.2%) to 19 mg/ml (~ 1.9%)
- 20 mg/ml (~ 2.0%) or more
- Do not know

For regression models, we categorized these as 19 mg/ml or less, 20 mg/ml or more or do not know.

#### Vaping frequency

Participants who vape were asked: ‘How many times per day on average do you use your nicotine replacement product or products?’. People who reported vaping at least once a day were considered to be vaping daily (versus non‐daily). Those who responded ‘do not know’ were excluded.

#### Time variables

Survey month (July 2016–January 2023) was included in our models to account for changes over the study period. We checked for seasonal differences in source of purchase using the Osborn, Chui, Smith & Birchenhall (OCSB) test. We did not detect any significant seasonality, so did not adjust for this in our models. We reported descriptive data by survey year (2016/2017, 2018/2019, 2020/2021, 2022/2023). We did not test for autocorrelation.

### Statistical analysis

The data used for these analyses are available on Open Science Framework (osf.io/85tk3/). The Smoking Toolkit Study uses raking to weight the sample to match the population in England on the dimensions of age, social grade, region, housing tenure, ethnicity and working status within sex. This profile is determined each month by combining data from the 2011 and 2021 UK Census, the Office for National Statistics mid‐year estimates and the annual National Readership Survey [4]. The following analyses used weighted data. Missing cases were excluded on a per‐analysis basis.

To address research question 1 (proportion buying from each source), we reported descriptive data on the proportion of people who vape who reported usually buying their vaping products from (a) vape shops, (b) supermarkets/convenience stores, (c) on‐line and (d) other sources.

To address research questions 2 and 3 (associations with user and device characteristics), we reported descriptive data on user and device characteristics stratified by source of purchase, overall and by survey year. We then used log‐binomial regression to analyse associations of sources of purchase (outcome variables, dummy‐coded as one‐versus‐rest—e.g. vape shops versus all other) with:

- Socio‐demographic characteristics and smoking status (unadjusted and in a multivariable model adjusting for survey month)
- main type of e‐cigarette used, usual nicotine concentration and vaping frequency (unadjusted and in a multivariable model adjusting for socio‐demographic characteristics, smoking status and survey month)

To address research question 4 (changes over time), we used log‐binomial regression to analyse associations of sources of purchase (outcome variables, dummy‐coded as one‐versus‐rest—e.g. vape shops versus all other) with survey month. For all regression analyses (i.e. those addressing research questions 2–4), survey month was modelled using restricted cubic splines to allow relationships with time to be flexible and non‐linear, while avoiding categorization. We decided a priori to use five knots when modelling survey month, as our study period was relatively long (7 years) and we wanted to allow sufficient flexibility to capture any shorter‐term fluctuations in source of purchase within this period. To explore changes in associations over time, we repeated the unadjusted models adding the interaction between survey month and each user and device characteristic. Each interaction was added to the unadjusted model in turn. We used these models to derive estimated modelled proportions buying from each source of purchase across the study period, which we plotted against unmodelled weighted monthly data points.

## RESULTS

Of 120 799 participants surveyed in eligible waves, 7128 (5.9%) reported current vaping. We excluded 621 participants with missing data on source of purchase (81 who responded ‘do not know’ and 540 who did not provide a response), leaving a final sample for analysis of 6507 people who vape [weighted descriptive statistics: mean (standard deviation) age = 41.0 (15.1) years, 44.6% women, 54.0% social grades C2DE]. Relative to the analysed sample, a higher proportion of the group who were excluded were aged < 25 (21.7 versus 15.4%), had never regularly smoked (13.0 versus 6.4%), mainly used pod devices (36.6 versus 15.2%), used no nicotine (24.7 versus 12.2%) or were unsure about their nicotine concentration (32.2 versus 5.0%) and vaped non‐daily (37.1 versus 23.3%) (Supporting information, Table S1).

### Proportion buying from each source (research question 1)

Overall, 37.3% [95% confidence intervals (CI) = 36.0–38.6%] of participants reported usually buying their vaping products from vape shops, 32.3% (31.1–33.6%) from supermarkets/convenience stores, 24.4% (23.3–25.6%) on‐line and 5.9% (5.3–6.6%) from other sources.

### Associations with user and device characteristics (research questions 2 and 3)

Table 1 shows the proportion buying from each source of purchase across the whole study period, by user and device characteristics. Table 2 summarizes independent associations of source of purchase with socio‐demographic characteristics and smoking status across the period, adjusted for survey month. Younger age groups were more likely to report buying from vape shops, while older age groups more commonly bought from supermarkets/convenience stores. Older age groups (in particular those aged ≥ 65) were more likely than younger groups to buy from other sources. Women were more likely than men to buy from supermarkets/convenience stores and less likely to buy on‐line. Those from more disadvantaged social grades (C2DE) were more likely than those from more advantaged social grades (ABC1) to buy from vape shops and other sources and less likely to buy on‐line. Relative to people who currently smoke, those who had never smoked were less likely to buy from vape shops and those who formerly smoked were less likely to buy from supermarkets/convenience stores. People who had formerly or never smoked were more likely than those who currently smoke to buy on‐line.

**TABLE 2:** Independent associations of usual source of purchase with socio‐demographic characteristics and smoking status.

	Vape shop		Supermarket/convenience store		On‐line		Other	
Characteristic	RR_adj_ (95% CI)	*P*	RR_adj_ (95% CI)	*P*	RR_adj_ (95% CI)	*P*	RR_adj_ (95% CI)	*P*
Age (years)								
18–24	–		–		–		–	
25–34	0.95 (0.86, 1.05)	0.306	1.00 (0.88, 1.13)	0.965	1.05 (0.90, 1.23)	0.526	1.20 (0.82, 1.77)	0.351
35–44	0.84 (0.75, 0.94)	0.003	1.04 (0.91, 1.19)	0.566	1.16 (0.99, 1.36)	0.075	1.16 (0.77, 1.75)	0.476
45–54	0.82 (0.73, 0.92)	< 0.001	1.12 (0.99, 1.27)	0.084	1.05 (0.89, 1.23)	0.557	1.45 (0.99, 2.13)	0.055
55–64	0.79 (0.70, 0.89)	< 0.001	1.11 (0.97, 1.28)	0.120	1.08 (0.91, 1.29)	0.353	1.49 (1.00, 2.22)	0.049
65+	0.69 (0.60, 0.80)	< 0.001	1.26 (1.09, 1.46)	0.001	0.98 (0.80, 1.19)	0.833	2.08 (1.40, 3.09)	< 0.001
Gender								
Men	–		–		–		–	
Women	1.06 (0.99, 1.13)	0.099	1.12 (1.04, 1.21)	0.003	0.82 (0.75, 0.89)	< 0.001	0.85 (0.69, 1.05)	0.129
Social grade								
ABC1	–		–		–		–	
C2DE	1.10 (1.03, 1.18)	0.004	1.05 (0.97, 1.13)	0.219	0.75 (0.68, 0.82)	< 0.001	1.38 (1.12, 1.70)	0.003
Smoking status								
Current smoking	–		–		–		–	
Former smoking	0.97 (0.90, 1.04)	0.333	0.79 (0.73, 0.86)	< 0.001	1.44 (1.31, 1.59)	< 0.001	0.98 (0.78, 1.23)	0.845
Never smoking	0.78 (0.67, 0.93)	0.004	0.96 (0.83, 1.12)	0.637	1.46 (1.22, 1.74)	< 0.001	1.15 (0.74, 1.77)	0.529

*Note*: See Supporting information, Table S6 for unadjusted associations.

Abbreviations: CI = confidence interval; RR_adj_, = relative risk adjusted for all characteristics in the table and survey month (modelled non‐linearly using restricted cubic splines with five knots).

Table 3 summarizes independent associations of source of purchase with device characteristics and frequency of vaping across the period, adjusted for socio‐demographic characteristics, smoking status and survey month. Relative to those who mainly used disposable e‐cigarettes, those who mainly used refillable and pod devices were more likely to buy from vape shops and on‐line and less likely to buy from supermarkets/convenience stores. Those who reported vaping daily were more likely than non‐daily users to buy from vape shops and less likely to buy from supermarkets/convenience stores. In unadjusted analyses (Supporting information, Table S7), those using nicotine concentrations above 20 mg/ml and those reporting that they did not know the nicotine concentration they were using were significantly more likely to buy from supermarkets/convenience stores than those using lower nicotine concentrations—notably, the proportion who did not know their nicotine concentration was twice as high among those buying from supermarkets/convenience stores (48.6%) than those buying from vape shops or on‐line (22.4 and 20.5%, respectively; Table 1). These differences were attenuated when we mutually adjusted for all e‐cigarette and socio‐demographic characteristics (Table 3).

**TABLE 3:** Independent associations of usual source of purchase with e‐cigarette device characteristics and vaping frequency.

Characteristic	Vape shop		Supermarket/convenience store		On‐line		Other	
	RR_adj_ (95% CI)	*P*	RR_adj_ (95% CI)	*P*	RR_adj_ (95% CI)	*P*	RR_adj_ (95% CI)	*P*
Device type								
Disposable	–		–		–		–	
Refillable	2.37 (1.92, 2.93)	< 0.001	0.43 (0.39, 0.48)	< 0.001	2.21 (1.67, 2.93)	< 0.001	0.67 (0.45, 1.00)	0.049
Pod	1.52 (1.20, 1.92)	< 0.001	0.78 (0.69, 0.87)	< 0.001	2.01 (1.48, 2.72)	< 0.001	0.52 (0.32, 0.83)	0.006
Nicotine concentration								
19 mg/mL or less	–		–		–		–	
20 mg/mL or more	0.99 (0.84, 1.16)	0.889	0.98 (0.85, 1.12)	0.730	0.98 (0.79, 1.21)	0.827	1.32 (0.82, 2.13)	0.259
Do not know	0.79 (0.63, 0.99)	0.043	1.08 (0.94, 1.24)	0.277	0.86 (0.66, 1.13)	0.278	1.58 (1.05, 2.38)	0.029
Frequency of vaping								
Non‐daily	–		–		–		–	
Daily	1.10 (1.01, 1.20)	0.031	0.94 (0.86, 1.02)	0.153	0.99 (0.88, 1.13)	0.924	0.80 (0.62, 1.03)	0.084

*Notes*: See Supporting information, Table S7 for unadjusted associations.

Abbreviations: CI, confidence interval; RR_adj_, relative risk adjusted for all characteristics in the table, age, gender, social grade, smoking status and survey month (modelled non‐linearly using restricted cubic splines with five knots).

### Time trends (research question 4)

**FIGURE 1:**
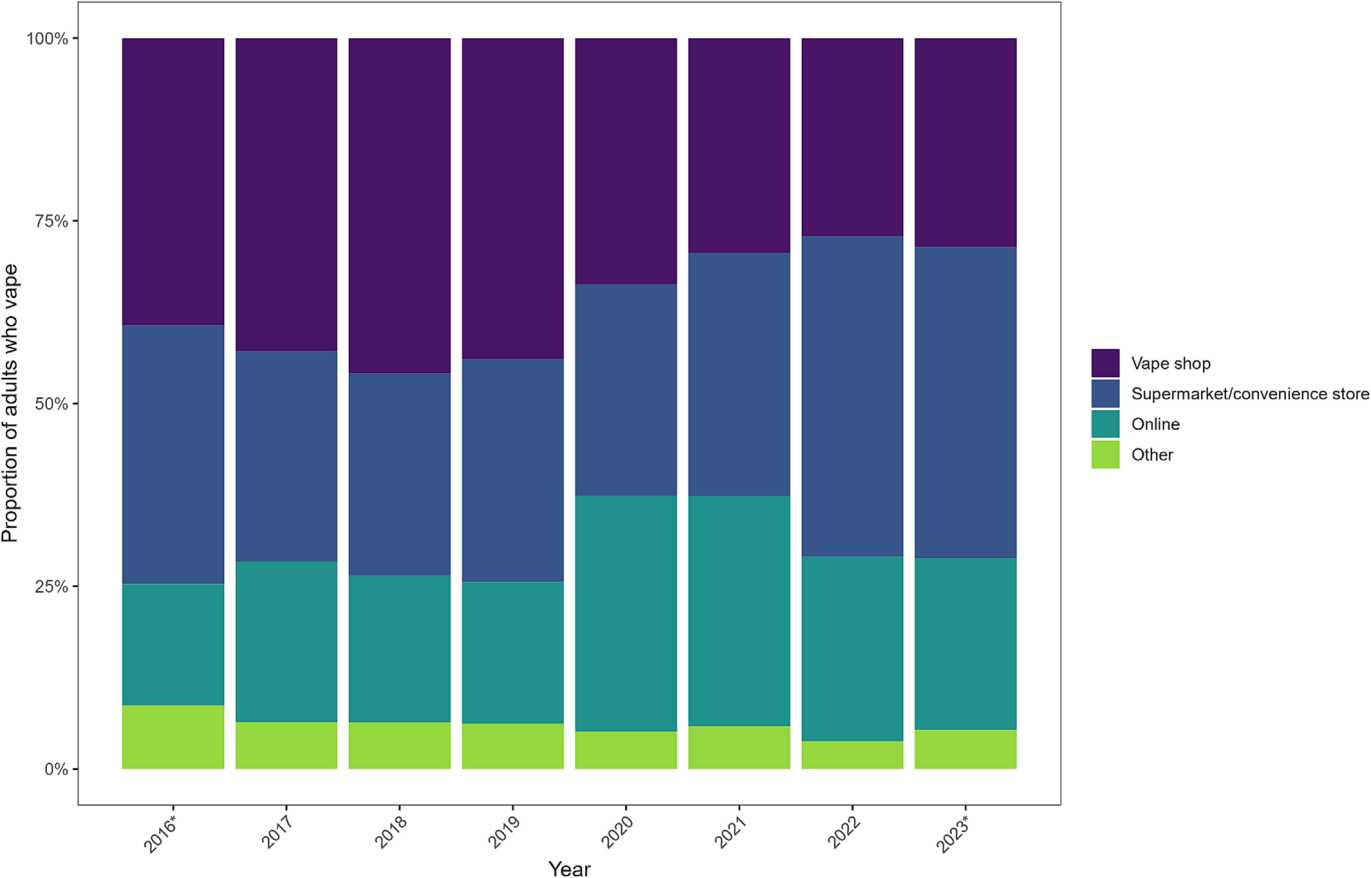
Usual source of purchase of vaping products among adults in England, by year. Data represent unadjusted weighted proportions. *2016 includes data from July to December only and 2023 includes data from January to April only.

There were significant changes over time regarding where people reported buying their vaping products from (Figure 1). Up to the start of 2020, vape shops were consistently the most common source of purchase (mean modelled monthly proportion, July 2016–December 2019 = 43.3%), followed by supermarkets/convenience stores (29.7%), on‐line retailers (20.5%) and other sources (6.6%).

**FIGURE 2:**
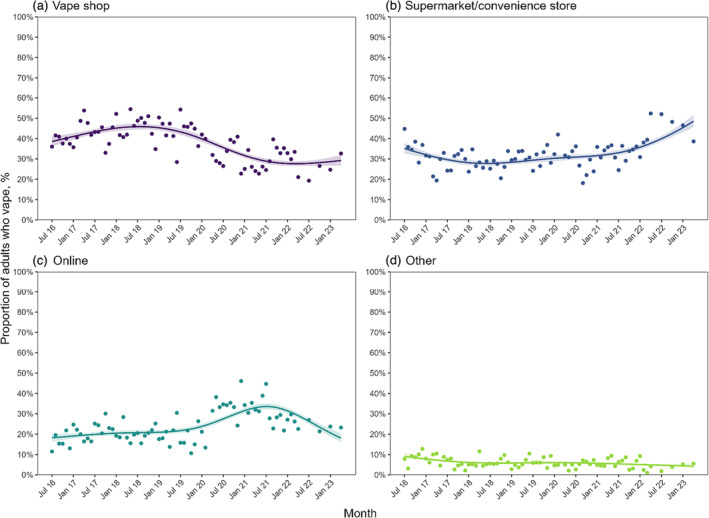
Time trends in the proportion of adults in England who vape reporting usually buying their vaping products from (a) vape shops, (b) supermarkets/convenience stores, (c) on‐line and (d) other sources, July 2016–April 2023. Lines represent modelled weighted prevalence by survey month, modelled non‐linearly using restricted cubic splines (five knots). Shaded bands represent standard errors. Points represent observed weighted prevalence by month.

Models indicated that the proportion buying their vaping products from vape shops peaked in August 2018, at 45.8% (95% CI = 43.3–48.5) and has been roughly stable at approximately 28% (with upper CI consistently less than 31%) since mid‐2021 (Figure 2a). The rise and subsequent fall in the proportion purchasing from vape shops up to and since mid‐2018 was most pronounced among younger adults (18–34 years) (Supporting information, Figure S1). There also appeared to be a rise since mid‐2021 in the proportion buying from vape shops among those who had formerly or never smoked and those who mainly used refillable or pod devices, while rates continued to fall among those who currently smoked and those who mainly used disposable e‐cigarettes, although these interactions were not statistically significant (Supporting information, Figure S1).

The proportion buying their vaping products from supermarkets/convenience stores rose significantly during 2021–23, from 31.6% (29.6–33.7%) in January 2021 to 48.5% (42.9–54.8%) in April 2023 (Figure 2b). This saw supermarkets/convenience stores overtake vape shops in February 2021 to become the most popular source of purchase. The rise was most pronounced among younger adults (18–35 years)—who went from the age group least likely to buy from supermarkets/convenience stores to the age group most likely to—and among those mainly using disposable devices (Supporting information, Figure S2). By April 2023, 59.6% (50.7–70.1%) of 18–35‐year‐olds and 77.5% (68.9–87.1%) of those mainly using disposables reported usually buying their vaping products from supermarkets/convenience stores. By contrast, the proportion of people mainly using pod devices buying from supermarkets/convenience stores fell from mid‐2021 (Supporting information, Figure S2), which may reflect a change in product choice from pods to disposables among people buying from these stores. There also appeared to be a more pronounced rise in the proportion who reported current smoking, using high‐strength nicotine e‐cigarettes (≥ 20 mg/ml) and potentially vaping non‐daily, buying their products from supermarkets/convenience stores—although interactions were not statistically significant (Supporting information, Figure S2).

The proportion buying their vaping products on‐line rose significantly to 33.6% (31.0–36.5%) in July 2021 before falling to 18.1% (14.4–22.7%) by April 2023 (Figure 2c). The rise was most pronounced among older adults (≥ 65 years) and men, and the fall occurred soonest among younger adults (18–34 years; Supporting information, Figure S3). Men were initially more likely to buy on‐line, and the rise and then decline was also more pronounced than among women and among those purchasing refillable devices (Supporting information, Figure S3).

Finally, the proportion buying their vaping products from other sources declined steadily over time, from 9.1% (6.6–12.7%) in July 2016 to 4.3% (2.6–7.1%) in April 2023 (Figure 2d), with no notable differences in trends by user or device characteristics (Supporting information, Figure S4).

## DISCUSSION

Adults in England buy vaping products from a variety of sources. Up to 2020, vape shops were the most common source of purchase (where more than four in 10 people who vape reported usually buying their vaping products), followed by supermarkets/convenience stores (three in 10) and on‐line retailers (two in 10). In 2020, these purchasing patterns shifted: on‐line purchasing increased and vape‐shop purchasing fell. The rise in on‐line purchasing was short‐lived and soon returned to baseline levels. However, the fall in vape‐shop purchasing persisted, displaced by a rapid rise in purchasing from supermarkets and convenience stores from 2021. Purchasing from other sources (e.g. cheap from friends) remains relatively rare (fewer than one in 10) and has declined over recent years.

These changes in trends are likely to be driven by two key factors. The first is the COVID‐19 pandemic. Social distancing measures introduced in 2020 to control the spread of the virus saw bricks‐and‐mortar vape shops forced to close under social distancing measures to control the spread of the virus, while on‐line retailers were able to operate as usual [9, 10]. This likely explains the fall in vape‐shop purchasing and rise in on‐line purchasing we observed in 2020, with on‐line purchasing returning to pre‐pandemic levels after restrictions on vape shops were lifted.

The second factor is the growing popularity of a new form of disposable e‐cigarette sold under brand names including ‘Puff Bar’, ‘Elf Bar’ or ‘Lost Mary’. Between 2021 and 2022, use of disposable vapes rose sharply in England as these new products rapidly became popular among young people [11, 12]. Not only did the timing of the rise in supermarket/convenience store purchasing coincide with this surge in use of disposable e‐cigarettes, but it was particularly pronounced among young adults and those who reported mainly using disposable e‐cigarettes. There was also some evidence the rise was greater among those using higher‐strength nicotine concentrations, which is consistent with the high‐concentration (20 mg/ml in EU/UK) nicotine salts e‐liquid in these new disposable devices [13], and those vaping non‐daily.

How to regulate new disposable e‐cigarettes to prevent uptake among young people who would not otherwise have smoked is a pressing issue in England and other nations within the United Kingdom and internationally. Our data show that supermarkets and convenience stores are currently the main source of disposable e‐cigarettes for young people and may therefore be the best target for regulation (e.g. point‐of‐sale display bans). However, such decisions should be considered carefully, as it is important that any measures to reduce uptake of vaping among young people who have never smoked do not inadvertently reduce the appeal of, or access to, e‐cigarettes as effective cessation aids and less harmful alternatives for people who smoke tobacco [1].

Strengths of this study include the nationally representative sample and monthly data collection over 7 years. However, there were some differences between the analysed sample and those who were excluded because they did not respond to the question on source of purchase. The excluded group seemed likely to be less established vapers, characterized by younger age, having never regularly smoked, vaping non‐daily and either using no nicotine or being unsure of their usual nicotine concentration. As a result, our results probably reflect the purchasing habits of more established vapers. Another key limitation was the change in mode of data collection from face‐to‐face to telephone interviews in April 2020, when the COVID‐19 pandemic began. Nonetheless, the two methods show good comparability [6, 7, 8], and there is no obvious reason to expect participants to respond differently to the question assessing usual source of purchase of vaping products. Another limitation was that participants were only asked about the places that they usually buy their vaping products from, not every place they buy from. It is likely that some people buy from multiple locations, meaning absolute prevalence of purchasing in those locations will be higher than our estimates. Tests of interactions with age were limited by the need to categorize people into broad age groups to provide enough data within each such that trends could be estimated with sufficient precision, and power was improved to test interactions. There was a degree of arbitrariness to the exact selection of different age groups, but we broadly selected the groups to distinguish between those at ages at greater risk of uptake (among never smokers) compared with using e‐cigarettes for cessation/harm reduction/relapse prevention in middle‐aged adults, compared with older adults for whom, thus far, e‐cigarettes have proved less popular. Finally, our analyses focused upon adults aged 18 years and older (due to availability of data), so our findings do not provide insight into where younger people, who cannot legally buy e‐cigarettes in England, acquire their vaping products.

In conclusion, supermarkets and convenience stores have recently overtaken vape shops to become the most popular source of purchase of vaping products in England. This is probably linked to the rising popularity of new disposable e‐cigarettes among young people, with rises in supermarket/convenience store purchasing more pronounced among people who are younger, mainly using disposable e‐cigarettes, and potentially those using higher‐strength devices and vaping non‐daily.

## AUTHOR CONTRIBUTIONS

**Sarah Jackson:** Conceptualization (equal); formal analysis (lead); investigation (equal); methodology (equal); visualization (lead); writing—original draft (lead). **Harry Tattan‐Birch:** Conceptualization (equal); investigation (equal); methodology (equal); visualization (supporting); writing—review and editing (equal). **Jamie Brown:** Conceptualization (equal); data curation (lead); funding acquisition (lead); investigation (equal); methodology (equal); supervision (lead); writing—review and editing (equal).

## DECLARATION OF INTERESTS

J.B. has received unrestricted research funding from Pfizer and J&J, who manufacture smoking cessation medications. All authors declare no financial links with tobacco companies, e‐cigarette manufacturers or their representatives.

## ETHICS STATEMENT

Ethical approval for the STS was granted originally by the UCL Ethics Committee (ID 0498/001). The data are not collected by UCL and are anonymized when received by UCL.

## Supporting information


**Table S1.** Characteristics of included (analysed) sample and excluded participants.
**Table S2.** Descriptive data on source of purchase, overall and by user and device characteristics, 2016/17 (*n* = 1523).
**Table S3.** Descriptive data on source of purchase, overall and by user and device characteristics, 2018/19 (*n* = 1995).
**Table S4.** Descriptive data on source of purchase, overall and by user and device characteristics, 2020/21 (*n* = 2027).
**Table S5.** Descriptive data on source of purchase, overall and by user and device characteristics, 2022/23 (*n* = 962).
**Table S6.** Unadjusted associations of usual source of purchase with sociodemographic characteristics and smoking status.
**Table S7.** Unadjusted associations of usual source of purchase with e‐cigarette device characteristics and vaping frequency.
**Figure S1.** Time trends in the proportion of adults in England who vape reporting usually buying their vaping products from vape shops by user and device characteristics, July 2016 to April 2023. Lines represent modelled weighted prevalence by survey month, modelled non‐linearly using restricted cubic splines (five knots). Shaded bands represent standard errors. Points represent observed weighted prevalence by month.
**Figure S2.** Time trends in the proportion of adults in England who vape reporting usually buying their vaping products from supermarkets/convenience stores by user and device characteristics, July 2016 to April 2023. Lines represent modelled weighted prevalence by survey month, modelled non‐linearly using restricted cubic splines (five knots). Shaded bands represent standard errors. Points represent observed weighted prevalence by month.
**Figure S3.** Time trends in the proportion of adults in England who vape reporting usually buying their vaping products on‐line by user and device characteristics, July 2016 to April 2023. Lines represent modelled weighted prevalence by survey month, modelled non‐linearly using restricted cubic splines (five knots). Shaded bands represent standard errors. Points represent observed weighted prevalence by month.
**Figure S4.** Time trends in the proportion of adults in England who vape reporting usually buying their vaping products from other sources by user and device characteristics, July 2016 to April 2023. Lines represent modelled weighted prevalence by survey month, modelled non‐linearly using restricted cubic splines (five knots). Shaded bands represent standard errors. Points represent observed weighted prevalence by month.

## Data Availability

Data are available on Open Science Framework (osf.io/85tk3).
