# Supplementary material for: Trends in where people buy their vaping products and differences by user and device characteristics: A population study in England, 2016–23
Source: Addiction. 2023 Nov 14;120(3):539–48. doi: 10.1111/add.16387 (PMC11813730; doi:10.1111/add.16387)
Supplement: Supplementary file 1 — Table S1. Characteristics of included (analysed) sample and excluded participants. Table S2. Descriptive data on source of purchase, overall and by user and device characteristics, 2016/17 (n = 1523). Table S3. Descriptive data on source of purchase, overall and by user and device characteristics, 2018/19 (n = 1995). Table S4. Descriptive data on source of purchase, overall and by user and device characteristics, 2020/21 (n = 2027). Table S5. Descriptive data on source of purchase, overall and by user and device characteristics, 2022/23 (n = 962). Table S6. Unadjusted associations of usual source of purchase with sociodemographic characteristics and smoking status. Table S7. Unadjusted associations of usual source of purchase with e‐cigarette device characteristics and vaping frequency. Figure S1. Time trends in the proportion of adults in England who vape reporting usually buying their vaping products from vape shops by user and device characteristics, July 2016 to April 2023. Lines represent modelled weighted prevalence by survey month, modelled non‐linearly using restricted cubic splines (five knots). Shaded bands represent standard errors. Points represent observed weighted prevalence by month. Figure S2. Time trends in the proportion of adults in England who vape reporting usually buying their vaping products from supermarkets/convenience stores by user and device characteristics, July 2016 to April 2023. Lines represent modelled weighted prevalence by survey month, modelled non‐linearly using restricted cubic splines (five knots). Shaded bands represent standard errors. Points represent observed weighted prevalence by month. Figure S3. Time trends in the proportion of adults in England who vape reporting usually buying their vaping products on‐line by user and device characteristics, July 2016 to April 2023. Lines represent modelled weighted prevalence by survey month, modelled non‐linearly using restricted cubic splines (five knots). Shaded bands represent st [file ADD-120-539-s001.docx]

### S1 Table. Characteristics of included (analysed) sample and excluded participants

|  | **Analysed**  **(*n*=6,507)^1^** | **Excluded**  **(*n*=621)^1^** |
| --- | --- | --- |
| Age (years) |  |  |
| 18-24 | 15.4% | 21.7% |
| 25-34 | 25.2% | 25.6% |
| 35-44 | 20.0% | 16.1% |
| 45-54 | 18.8% | 15.6% |
| 55-64 | 12.6% | 12.2% |
| 65+ | 8.0% | 8.8% |
|  |  |  |
| Gender |  |  |
| Men | 55.0% | 53.5% |
| Women | 44.6% | 45.5% |
| In another way | 0.4% | 1.0% |
|  |  |  |
| Social grade C2DE |  |  |
| ABC1 | 46.0% | 45.8% |
| C2DE | 54.0% | 54.2% |
|  |  |  |
| Smoking status |  |  |
| Never smoking | 6.4% | 13.0% |
| Former smoking | 39.9% | 31.3% |
| Current smoking | 53.8% | 55.8% |
|  |  |  |
| Device type |  |  |
| Disposable | 9.2% | 4.6% |
| Refillable | 75.6% | 58.7% |
| Pod | 15.2% | 36.6% |
|  |  |  |
| Nicotine concentration |  |  |
| No nicotine | 12.2% | 24.7% |
| 6 mg/ml or less | 41.6% | 26.9% |
| 7-11 mg/ml | 11.7% | 9.4% |
| 12-19 mg/ml | 22.4% | 5.8% |
| 20 mg/ml or more | 7.1% | 1.0% |
| Don’t know | 5.0% | 32.2% |
|  |  |  |
| Frequency of vaping |  |  |
| Non-daily | 23.3% | 37.1% |
| Daily | 76.7% | 62.9% |

^1^ Unweighted sample size.

Data shown are weighted column percentages. Note there were some missing data for some variables (among analysed/excluded participants, respectively: gender *n*=1/0; device type *n*=64/571; nicotine concentration *n*=59/559; frequency of vaping *n*=692/327) so numbers do not sum to the total sample size for these variables. Valid percentages are shown for ease of interpretation.

### S2 Table. Descriptive data on source of purchase, overall and by user and device characteristics, 2016/17 (*n*=1,523)

|  |  | **Usual source of purchase of vaping products** | | | |
| --- | --- | --- | --- | --- | --- |
|  | **Unweighted *N*** | **Vape shop** | **Supermarket/**  **convenience store** | **Online** | **Other** |
| Overall | 1523 | 41.7% | 30.8% | 20.3% | 7.1% |
|  |  |  |  |  |  |
| Age (years) |  |  |  |  |  |
| 18-24 | 219 | 50.0% | 24.7% | 20.7% | 4.7% |
| 25-34 | 300 | 47.4% | 26.2% | 20.0% | 6.5% |
| 35-44 | 291 | 40.4% | 29.5% | 23.2% | 6.9% |
| 45-54 | 300 | 37.8% | 33.9% | 20.3% | 8.0% |
| 55-64 | 237 | 37.9% | 35.8% | 18.1% | 8.2% |
| 65+ | 176 | 32.0% | 41.9% | 16.4% | 9.7% |
|  |  |  |  |  |  |
| Gender |  |  |  |  |  |
| Men | 823 | 39.2% | 27.3% | 25.6% | 7.8% |
| Women | 699 | 44.9% | 35.1% | 13.8% | 6.2% |
| In another way | 1 | 0.0% | 100.0% | 0.0% | 0.0% |
|  |  |  |  |  |  |
| Social grade C2DE |  |  |  |  |  |
| ABC1 | 767 | 41.0% | 28.4% | 25.1% | 5.4% |
| C2DE | 756 | 42.4% | 32.9% | 16.1% | 8.6% |
|  |  |  |  |  |  |
| Smoking status |  |  |  |  |  |
| Never smoking | 85 | 35.8% | 31.5% | 26.7% | 6.1% |
| Former smoking | 523 | 40.4% | 28.7% | 24.2% | 6.7% |
| Current smoking | 915 | 43.1% | 32.0% | 17.4% | 7.5% |
|  |  |  |  |  |  |
| Device type |  |  |  |  |  |
| Disposable | 87 | 21.1% | 56.7% | 9.4% | 12.7% |
| Refillable | 1,173 | 46.0% | 26.1% | 21.3% | 6.6% |
| Pod | 251 | 28.9% | 44.6% | 19.7% | 6.7% |
|  |  |  |  |  |  |
| Nicotine concentration |  |  |  |  |  |
| No nicotine | 196 | 35.4% | 31.9% | 21.3% | 11.5% |
| 6 mg/ml or less | 580 | 46.1% | 24.3% | 24.3% | 5.3% |
| 7-11 mg/ml | 218 | 38.6% | 39.8% | 15.3% | 6.2% |
| 12-19 mg/ml | 388 | 43.4% | 32.5% | 16.9% | 7.2% |
| 20 mg/ml or more | 67 | 36.3% | 32.6% | 24.4% | 6.6% |
| Don’t know | 60 | 29.6% | 41.8% | 16.2% | 12.3% |
|  |  |  |  |  |  |
| Frequency of vaping |  |  |  |  |  |
| Non-daily | 396 | 37.6% | 38.1% | 16.9% | 7.5% |
| Daily | 1,015 | 44.0% | 28.5% | 21.3% | 6.2% |
| Data shown are weighted row percentages. Note there were some missing data for some variables (device type *n*=12; nicotine concentration *n*=14; frequency of vaping *n*=112) so unweighted sample sizes do not sum to the total sample size for these variables. Valid percentages are shown for ease of interpretation. | | | | | |

### S3 Table. Descriptive data on source of purchase, overall and by user and device characteristics, 2018/19 (*n*=1,995)

|  |  | **Usual source of purchase of vaping products** | | | |
| --- | --- | --- | --- | --- | --- |
|  | **Unweighted *N*** | **Vape shop** | **Supermarket/**  **convenience store** | **Online** | **Other** |
| Overall | 1995 | 45.0% | 29.0% | 19.8% | 6.3% |
|  |  |  |  |  |  |
| Age (years) |  |  |  |  |  |
| 18-24 | 274 | 54.1% | 18.8% | 23.1% | 4.0% |
| 25-34 | 434 | 51.6% | 24.6% | 18.8% | 4.9% |
| 35-44 | 354 | 39.9% | 30.8% | 22.7% | 6.7% |
| 45-54 | 377 | 42.1% | 32.4% | 19.0% | 6.5% |
| 55-64 | 330 | 42.4% | 31.4% | 19.7% | 6.5% |
| 65+ | 226 | 35.6% | 40.2% | 12.5% | 11.7% |
|  |  |  |  |  |  |
| Gender |  |  |  |  |  |
| Men | 1,121 | 44.8% | 27.1% | 21.3% | 6.8% |
| Women | 870 | 45.4% | 31.4% | 17.6% | 5.6% |
| In another way | 4 | 26.0% | 51.0% | 23.1% | 0.0% |
|  |  |  |  |  |  |
| Social grade C2DE |  |  |  |  |  |
| ABC1 | 1,022 | 42.3% | 28.3% | 23.9% | 5.5% |
| C2DE | 973 | 47.2% | 29.6% | 16.4% | 6.9% |
|  |  |  |  |  |  |
| Smoking status |  |  |  |  |  |
| Never smoking | 110 | 41.9% | 23.3% | 28.5% | 6.4% |
| Former smoking | 713 | 45.3% | 24.9% | 23.4% | 6.5% |
| Current smoking | 1,172 | 45.1% | 32.2% | 16.6% | 6.1% |
|  |  |  |  |  |  |
| Device type |  |  |  |  |  |
| Disposable | 99 | 25.6% | 51.3% | 12.1% | 11.0% |
| Refillable | 1,535 | 47.5% | 25.1% | 21.1% | 6.3% |
| Pod | 337 | 39.4% | 39.6% | 16.0% | 5.0% |
|  |  |  |  |  |  |
| Nicotine concentration |  |  |  |  |  |
| No nicotine | 283 | 37.0% | 35.4% | 21.5% | 6.1% |
| 6 mg/ml or less | 876 | 51.4% | 20.4% | 22.9% | 5.2% |
| 7-11 mg/ml | 251 | 36.8% | 41.7% | 14.7% | 6.8% |
| 12-19 mg/ml | 445 | 42.0% | 33.4% | 17.7% | 6.9% |
| 20 mg/ml or more | 77 | 48.8% | 29.2% | 10.9% | 11.1% |
| Don’t know | 50 | 29.4% | 45.7% | 13.3% | 11.6% |
|  |  |  |  |  |  |
| Frequency of vaping |  |  |  |  |  |
| Non-daily | 443 | 39.5% | 34.9% | 18.6% | 7.0% |
| Daily | 1,409 | 47.6% | 26.6% | 19.9% | 5.9% |
| Data shown are weighted row percentages. Note there was some missing data for some variables (device type *n*=24; nicotine concentration *n*=13; frequency of vaping *n*=143) so numbers do not sum to the total sample size for these variables. Valid percentages are shown for ease of interpretation. | | | | | |

### S4 Table. Descriptive data on source of purchase, overall and by user and device characteristics, 2020/21 (*n*=2,027)

|  |  | **Usual source of purchase of vaping products** | | | |
| --- | --- | --- | --- | --- | --- |
|  | **Unweighted *N*** | **Vape shop** | **Supermarket/**  **convenience store** | **Online** | **Other** |
| Overall | 2027 | 31.3% | 31.3% | 31.8% | 5.5% |
|  |  |  |  |  |  |
| Age (years) |  |  |  |  |  |
| 18-24 | 313 | 35.6% | 31.6% | 27.9% | 4.9% |
| 25-34 | 479 | 31.2% | 29.5% | 33.5% | 5.8% |
| 35-44 | 350 | 33.6% | 28.2% | 34.9% | 3.3% |
| 45-54 | 380 | 29.2% | 33.5% | 31.4% | 5.9% |
| 55-64 | 296 | 27.7% | 35.5% | 30.4% | 6.4% |
| 65+ | 209 | 28.2% | 33.7% | 29.7% | 8.5% |
|  |  |  |  |  |  |
| Gender |  |  |  |  |  |
| Men | 1,075 | 30.8% | 29.8% | 33.7% | 5.7% |
| Women | 939 | 32.0% | 33.2% | 29.6% | 5.2% |
| In another way | 13 | 30.8% | 38.5% | 23.1% | 7.7% |
|  |  |  |  |  |  |
| Social grade C2DE |  |  |  |  |  |
| ABC1 | 1,138 | 28.4% | 30.3% | 37.1% | 4.2% |
| C2DE | 889 | 33.8% | 32.3% | 27.3% | 6.7% |
|  |  |  |  |  |  |
| Smoking status |  |  |  |  |  |
| Never smoking | 116 | 21.9% | 34.4% | 37.7% | 5.9% |
| Former smoking | 913 | 26.9% | 29.3% | 38.0% | 5.9% |
| Current smoking | 998 | 36.2% | 32.8% | 25.8% | 5.2% |
|  |  |  |  |  |  |
| Device type |  |  |  |  |  |
| Disposable | 104 | 19.1% | 57.9% | 14.8% | 8.2% |
| Refillable | 1,584 | 35.8% | 25.1% | 33.4% | 5.6% |
| Pod | 320 | 12.7% | 53.6% | 30.0% | 3.7% |
|  |  |  |  |  |  |
| Nicotine concentration |  |  |  |  |  |
| No nicotine | 238 | 34.5% | 22.5% | 34.5% | 8.5% |
| 6 mg/ml or less | 831 | 36.9% | 23.4% | 34.7% | 5.0% |
| 7-11 mg/ml | 200 | 22.8% | 41.2% | 31.1% | 4.9% |
| 12-19 mg/ml | 465 | 28.4% | 41.1% | 25.7% | 4.7% |
| 20 mg/ml or more | 108 | 22.7% | 33.3% | 38.9% | 5.1% |
| Don’t know | 164 | 21.8% | 46.7% | 25.4% | 6.1% |
|  |  |  |  |  |  |
| Frequency of vaping |  |  |  |  |  |
| Non-daily | 372 | 31.2% | 32.4% | 29.6% | 6.8% |
| Daily | 1,378 | 31.6% | 31.5% | 32.0% | 4.8% |
| Data shown are weighted row percentages. Note there was some missing data for some variables (device type *n*=19; nicotine concentration *n*=21; frequency of vaping *n*=277) so numbers do not sum to the total sample size for these variables. Valid percentages are shown for ease of interpretation. | | | | | |

### S5 Table. Descriptive data on source of purchase, overall and by user and device characteristics, 2022/23 (*n*=962)

|  |  | **Usual source of purchase of vaping products** | | | |
| --- | --- | --- | --- | --- | --- |
|  | **Unweighted *N*** | **Vape shop** | **Supermarket/**  **convenience store** | **Online** | **Other** |
| Overall | 962 | 27.6% | 43.5% | 24.7% | 4.3% |
|  |  |  |  |  |  |
| Age (years) |  |  |  |  |  |
| 18-24 | 214 | 26.0% | 53.3% | 16.2% | 4.5% |
| 25-34 | 240 | 28.5% | 47.8% | 20.0% | 3.7% |
| 35-44 | 165 | 27.7% | 39.3% | 29.3% | 3.7% |
| 45-54 | 162 | 33.6% | 32.9% | 28.7% | 4.8% |
| 55-64 | 117 | 24.0% | 35.9% | 34.9% | 5.2% |
| 65+ | 64 | 20.2% | 38.3% | 36.7% | 4.8% |
|  |  |  |  |  |  |
| Gender |  |  |  |  |  |
| Men | 495 | 27.2% | 42.9% | 26.2% | 3.7% |
| Women | 454 | 28.1% | 43.4% | 23.6% | 5.0% |
| In another way | 12 | 25.0% | 66.7% | 8.3% | 0.0% |
|  |  |  |  |  |  |
| Social grade C2DE |  |  |  |  |  |
| ABC1 | 573 | 25.5% | 43.3% | 26.6% | 4.7% |
| C2DE | 389 | 29.4% | 43.6% | 23.1% | 3.9% |
|  |  |  |  |  |  |
| Smoking status |  |  |  |  |  |
| Never smoking | 97 | 19.2% | 47.1% | 27.2% | 6.5% |
| Former smoking | 428 | 31.1% | 33.9% | 31.6% | 3.5% |
| Current smoking | 437 | 26.2% | 51.8% | 17.5% | 4.5% |
|  |  |  |  |  |  |
| Device type |  |  |  |  |  |
| Disposable | 301 | 11.8% | 73.1% | 10.5% | 4.6% |
| Refillable | 535 | 38.3% | 25.8% | 32.4% | 3.4% |
| Pod | 117 | 19.5% | 49.9% | 24.8% | 5.8% |
|  |  |  |  |  |  |
| Nicotine concentration |  |  |  |  |  |
| No nicotine | 98 | 33.9% | 33.5% | 28.3% | 4.2% |
| 6 mg/ml or less | 330 | 31.9% | 39.8% | 25.6% | 2.7% |
| 7-11 mg/ml | 77 | 21.8% | 44.1% | 29.2% | 4.9% |
| 12-19 mg/ml | 160 | 28.5% | 34.3% | 34.6% | 2.6% |
| 20 mg/ml or more | 203 | 24.1% | 54.7% | 15.2% | 6.0% |
| Don’t know | 83 | 15.1% | 58.3% | 17.8% | 8.8% |
|  |  |  |  |  |  |
| Frequency of vaping |  |  |  |  |  |
| Non-daily | 151 | 26.6% | 49.9% | 18.2% | 5.4% |
| Daily | 651 | 27.3% | 43.0% | 25.6% | 4.1% |
| Data shown are weighted row percentages. Note there was some missing data for some variables (gender *n*=1; device type *n*=9; nicotine concentration *n*=11; frequency of vaping *n*=160) so numbers do not sum to the total sample size for these variables. Valid percentages are shown for ease of interpretation. | | | | | |

### S6 Table. Unadjusted associations of usual source of purchase with sociodemographic characteristics and smoking status

|  | **Vape shop** | |  | **Supermarket/**  **convenience store** | |  | **Online** | |  | **Other** | |
| --- | --- | --- | --- | --- | --- | --- | --- | --- | --- | --- | --- |
| **Characteristic** | **RR (95% CI)** | ***p*** |  | **RR (95% CI)** | ***p*** |  | **RR (95% CI)** | ***p*** |  | **RR (95% CI)** | ***p*** |
| Age |  |  |  |  |  |  |  |  |  |  |  |
| 18-24 | — |  |  | — |  |  | — |  |  | — |  |
| 25-34 | 0.98 (0.88, 1.09) | 0.677 |  | 0.95 (0.84, 1.08) | 0.443 |  | 1.07 (0.92, 1.26) | 0.373 |  | 1.18 (0.80, 1.73) | 0.410 |
| 35-44 | 0.89 (0.79, 0.99) | 0.037 |  | 0.97 (0.85, 1.11) | 0.659 |  | 1.22 (1.04, 1.43) | 0.014 |  | 1.15 (0.76, 1.72) | 0.507 |
| 45-54 | 0.88 (0.79, 0.99) | 0.030 |  | 1.04 (0.91, 1.18) | 0.546 |  | 1.07 (0.91, 1.26) | 0.422 |  | 1.43 (0.98, 2.09) | 0.063 |
| 55-64 | 0.84 (0.75, 0.95) | 0.006 |  | 1.08 (0.94, 1.23) | 0.291 |  | 1.08 (0.91, 1.28) | 0.355 |  | 1.47 (0.99, 2.17) | 0.054 |
| 65+ | 0.75 (0.65, 0.86) | <0.001 |  | 1.20 (1.04, 1.38) | 0.012 |  | 0.96 (0.79, 1.17) | 0.695 |  | 2.07 (1.40, 3.06) | <0.001 |
|  |  |  |  |  |  |  |  |  |  |  |  |
| Gender |  |  |  |  |  |  |  |  |  |  |  |
| Men | — |  |  | — |  |  | — |  |  | — |  |
| Women | 1.05 (0.98, 1.12) | 0.200 |  | 1.15 (1.07, 1.24) | <0.001 |  | 0.79 (0.72, 0.87) | <0.001 |  | 0.88 (0.72, 1.09) | 0.256 |
|  |  |  |  |  |  |  |  |  |  |  |  |
| Social grade |  |  |  |  |  |  |  |  |  |  |  |
| ABC1 | — |  |  | — |  |  | — |  |  | — |  |
| C2DE | 1.12 (1.05, 1.20) | 0.001 |  | 1.07 (0.99, 1.15) | 0.092 |  | 0.72 (0.66, 0.79) | <0.001 |  | 1.37 (1.11, 1.69) | 0.003 |
|  |  |  |  |  |  |  |  |  |  |  |  |
| Smoking status |  |  |  |  |  |  |  |  |  |  |  |
| Current smoking | — |  |  | — |  |  | — |  |  | — |  |
| Former smoking | 0.90 (0.83, 0.96) | 0.003 |  | 0.82 (0.76, 0.89) | <0.001 |  | 1.53 (1.39, 1.68) | <0.001 |  | 0.97 (0.78, 1.21) | 0.803 |
| Never smoking | 0.74 (0.63, 0.88) | <0.001 |  | 0.98 (0.84, 1.14) | 0.810 |  | 1.54 (1.29, 1.84) | <0.001 |  | 1.04 (0.67, 1.60) | 0.868 |

RR, relative risk. CI, confidence interval.

### S7 Table. Unadjusted associations of usual source of purchase with e-cigarette device characteristics and vaping frequency

|  | **Vape shop** | |  | **Supermarket/**  **convenience store** | |  | **Online** | |  | **Other** | |
| --- | --- | --- | --- | --- | --- | --- | --- | --- | --- | --- | --- |
| **Characteristic** | **RR (95% CI)** | ***p*** |  | **RR (95% CI)** | ***p*** |  | **RR (95% CI)** | ***p*** |  | **RR (95% CI)** | ***p*** |
| Device type |  |  |  |  |  |  |  |  |  |  |  |
| Disposable | — |  |  | — |  |  | — |  |  | — |  |
| Refillable | 2.53 (2.09, 3.06) | <0.001 |  | 0.39 (0.36, 0.43) | <0.001 |  | 2.32 (1.82, 2.96) | <0.001 |  | 0.78 (0.56, 1.08) | 0.136 |
| Pod | 1.56 (1.25, 1.94) | <0.001 |  | 0.72 (0.66, 0.79) | <0.001 |  | 1.95 (1.49, 2.56) | <0.001 |  | 0.68 (0.45, 1.03) | 0.070 |
|  |  |  |  |  |  |  |  |  |  |  |  |
| Nicotine concentration |  |  |  |  |  |  |  |  |  |  |  |
| 19 mg/ml or less | — |  |  | — |  |  | — |  |  | — |  |
| 20 mg/ml or more | 0.75 (0.64, 0.88) | <0.001 |  | 1.38 (1.22, 1.57) | <0.001 |  | 0.88 (0.73, 1.07) | 0.196 |  | 1.17 (0.77, 1.78) | 0.450 |
| Don’t know | 0.58 (0.46, 0.72) | <0.001 |  | 1.59 (1.41, 1.81) | <0.001 |  | 0.82 (0.65, 1.04) | 0.095 |  | 1.50 (1.02, 2.20) | 0.038 |
|  |  |  |  |  |  |  |  |  |  |  |  |
| Frequency of vaping |  |  |  |  |  |  |  |  |  |  |  |
| Non-daily | — |  |  | — |  |  | — |  |  | — |  |
| Daily | 1.10 (1.01, 1.20) | 0.030 |  | 0.84 (0.77, 0.92) | <0.001 |  | 1.18 (1.04, 1.34) | 0.009 |  | 0.78 (0.61, 1.00) | 0.047 |

RR, relative risk. CI, confidence interval.

**
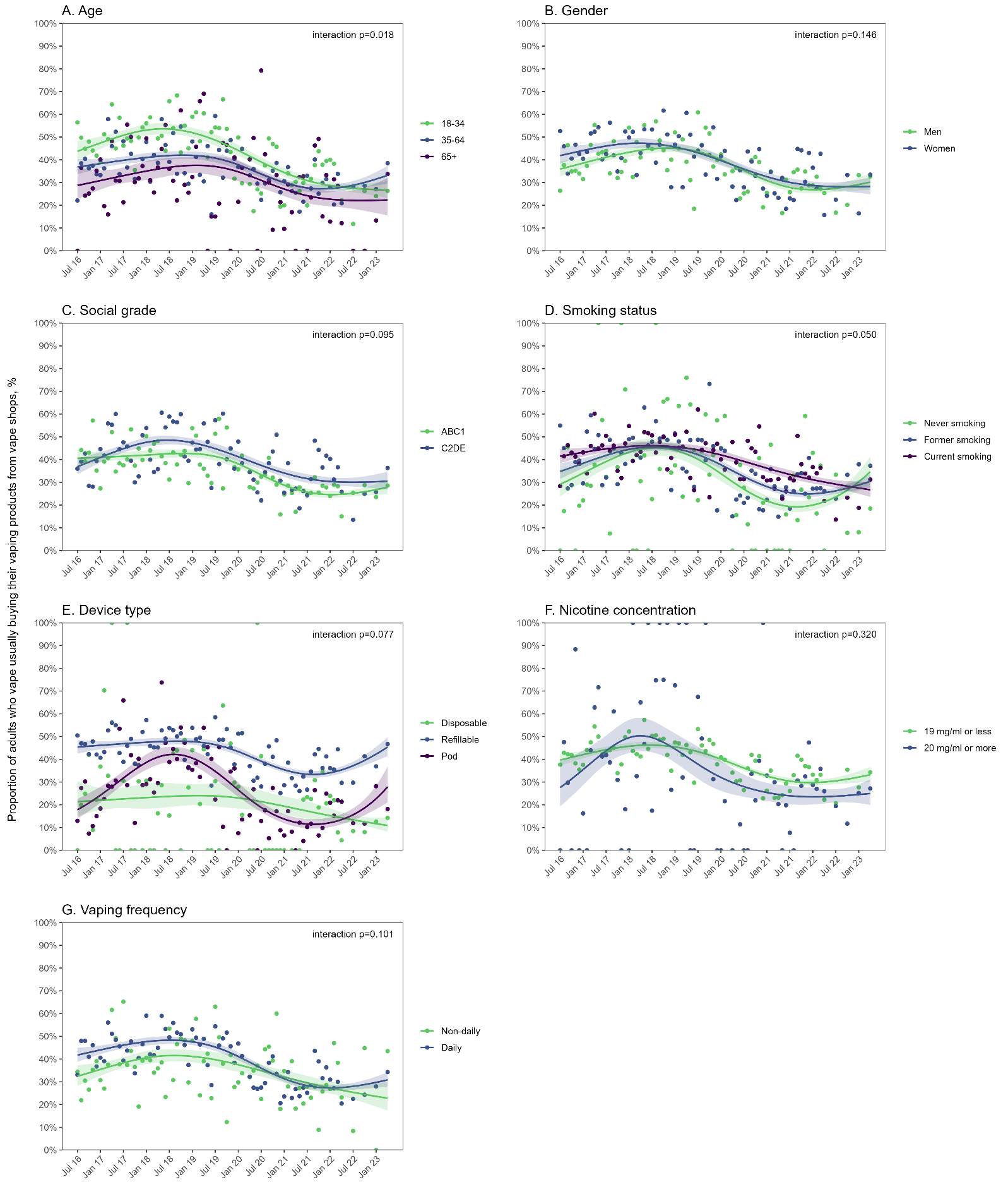
**

### S1 Figure. Time trends in the proportion of adults in England who vape reporting usually buying their vaping products from vape shops by user and device characteristics, July 2016 to April 2023. Lines represent modelled weighted prevalence by survey month, modelled non-linearly using restricted cubic splines (five knots). Shaded bands represent standard errors. Points represent observed weighted prevalence by month.

**
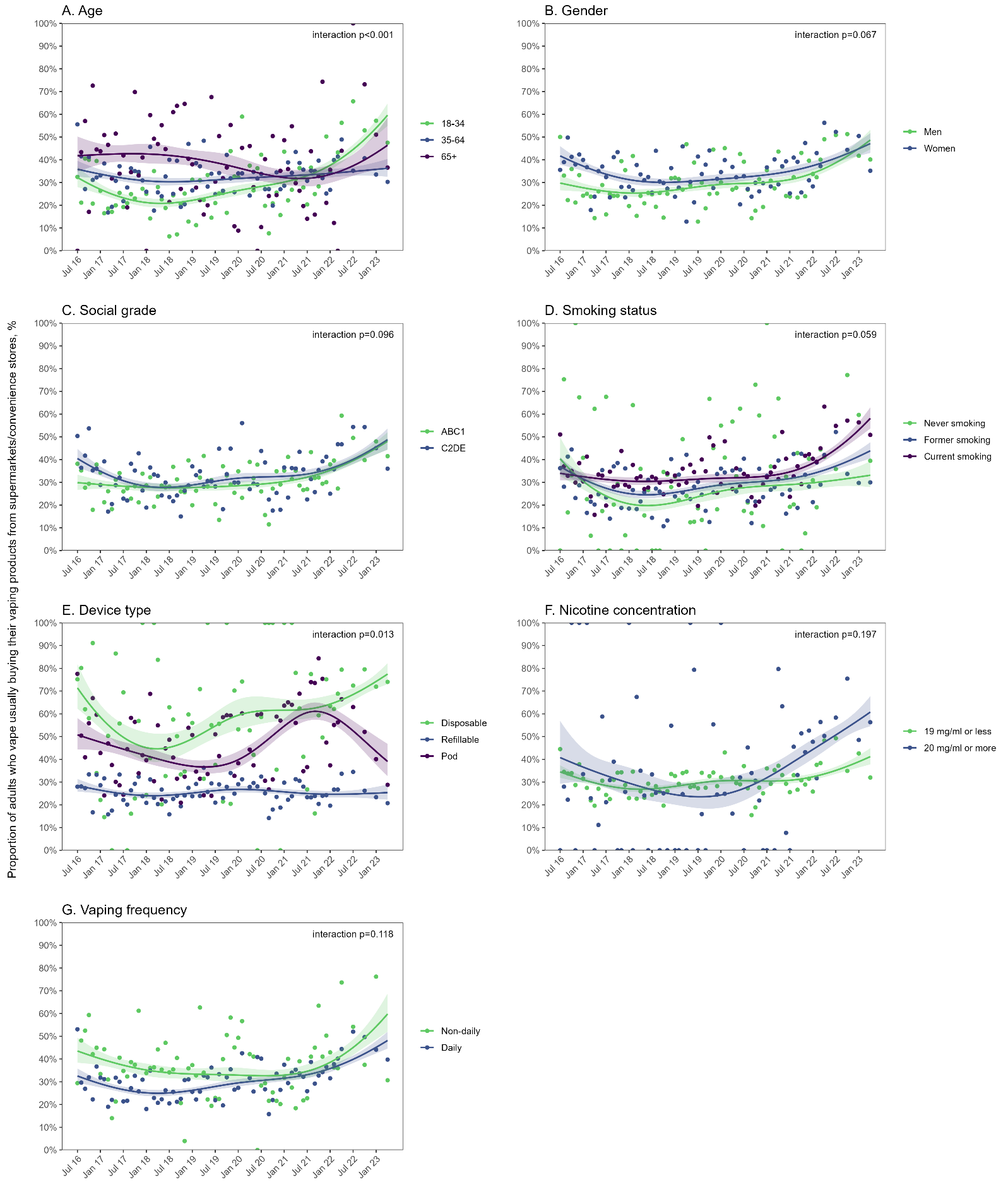
**

### S2 Figure. Time trends in the proportion of adults in England who vape reporting usually buying their vaping products from supermarkets/convenience stores by user and device characteristics, July 2016 to April 2023. Lines represent modelled weighted prevalence by survey month, modelled non-linearly using restricted cubic splines (five knots). Shaded bands represent standard errors. Points represent observed weighted prevalence by month.

**
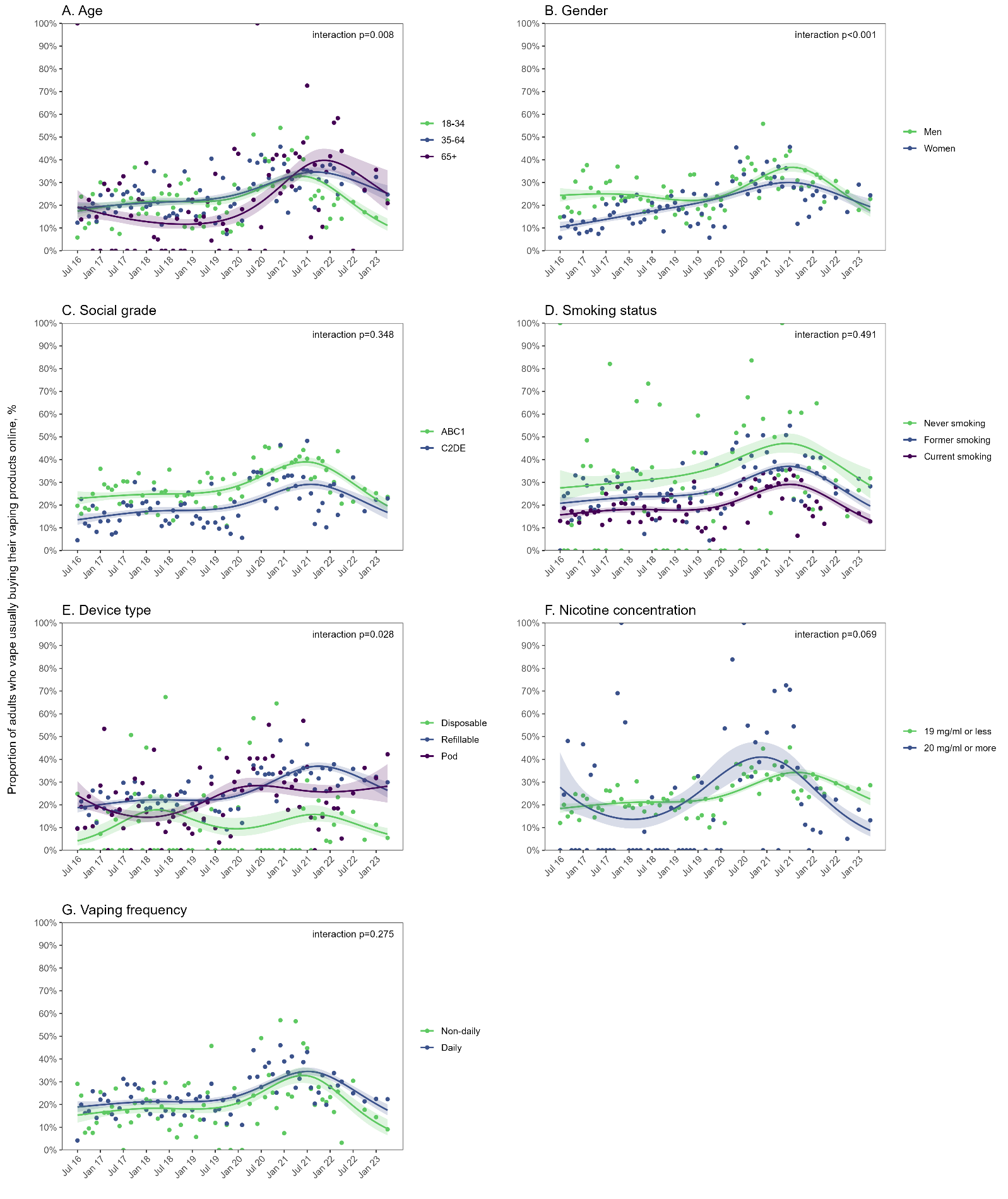
**

### S3 Figure. Time trends in the proportion of adults in England who vape reporting usually buying their vaping products online by user and device characteristics, July 2016 to April 2023. Lines represent modelled weighted prevalence by survey month, modelled non-linearly using restricted cubic splines (five knots). Shaded bands represent standard errors. Points represent observed weighted prevalence by month.

**
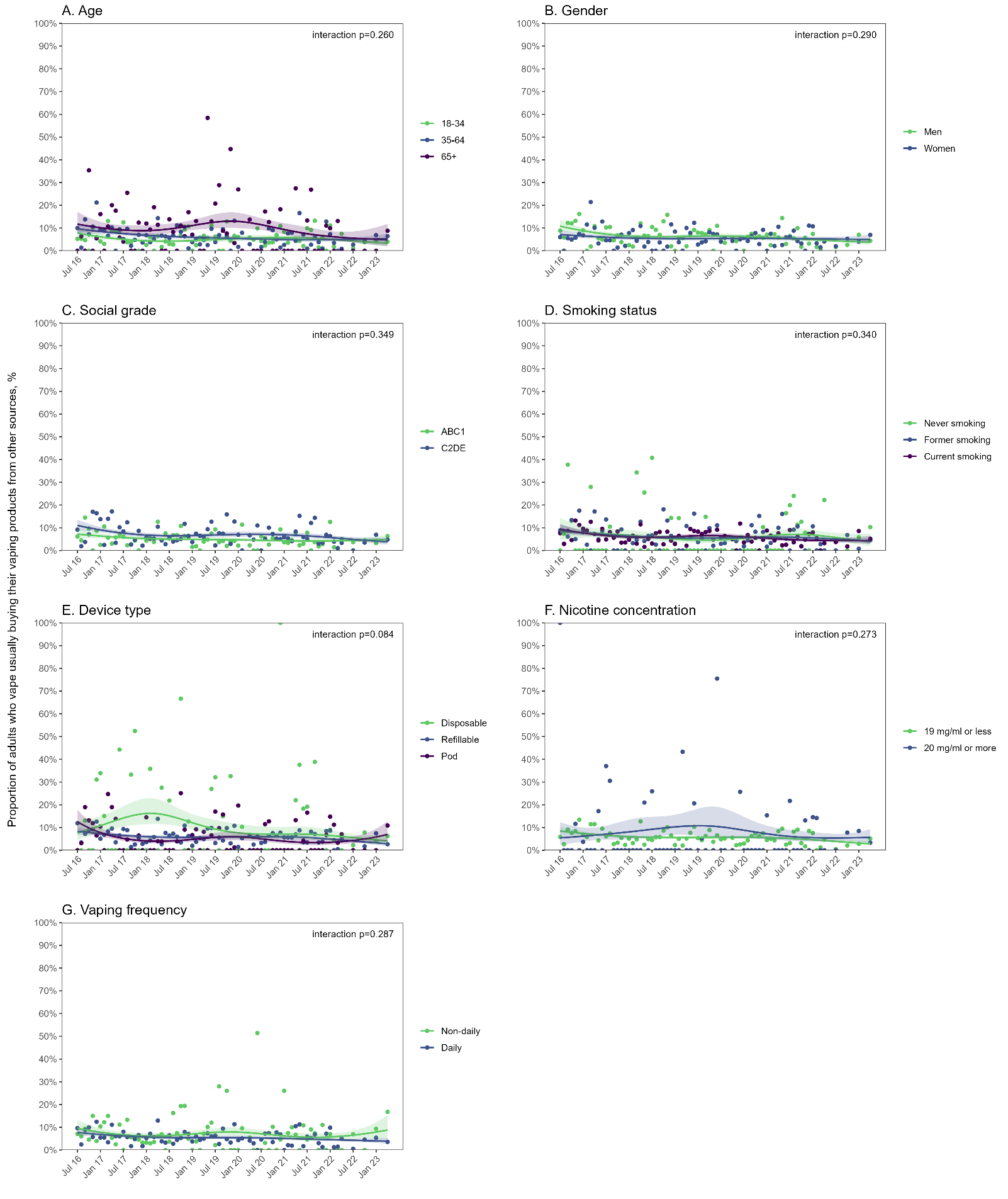
**

### S4 Figure. Time trends in the proportion of adults in England who vape reporting usually buying their vaping products from other sources by user and device characteristics, July 2016 to April 2023. Lines represent modelled weighted prevalence by survey month, modelled non-linearly using restricted cubic splines (five knots). Shaded bands represent standard errors. Points represent observed weighted prevalence by month.
